# Transitional initiatives for advancing the phasing out of the use of animals for drug and chemical safety testing: The IHI VICT3R project for reducing the use of animals by implementing virtual control groups

**DOI:** 10.1016/j.namjnl.2025.100065

**Published:** 2025-10-22

**Authors:** Ferran Sanz, Julia Matyjasiak, Javier Orihuel, Colleen M. Pike, Inari Soininen, Frank Bringezu, Olga Valverde, Mathieu Vinken, Janette Turner, Thomas Steger-Hartmann

**Affiliations:** a Universitat Pompeu Fabra, Barcelona, Spain; b Hospital del Mar Research Institute, Barcelona, Spain; c Vrije Universiteit Brussel, Brussels, Belgium; d Humane World for Animals, Washington, DC, United States of America; e SYNAPSE Research Management Partners SL, Madrid, Spain; f Merck Healthcare KGaA, Darmstadt, Germany; g Bayer AG, Pharmaceuticals, Berlin, Germany

**Keywords:** New approach methodologies, Reduction of animal use, Virtual control groups, Data sharing, *In silico* approaches

## Abstract

Despite rapid advances in human-relevant assay technologies and other innovative *in vitro* and *in silico* approaches, the safety assessment of chemicals and pharmaceutical drugs continues to heavily rely on animal testing, in part due to slow regulatory adaptation and a lack of transitional strategies that clear the way for the phasing out of animal testing while demonstrating the preservation of current safety standards. To overcome this hurdle, several international efforts are currently focused on systematically integrating new approach methodologies (NAMs) into regulatory frameworks with the goal of phasing out animal testing in the safety assessments. The present paper highlights the need for pragmatic transitional initiatives that reduce animal use while preserving scientific rigor and regulatory confidence by focusing on the IHI VICT3R project, a collaborative effort aimed at reducing animal use in safety assessments of drugs and chemicals by replacing concurrent control groups with virtual control groups (VCGs). The VICT3R project focuses on refining and implementing the VCG methodology, particularly in the standardization, curation and adequate matching of historical control data (HCD), as well as regulatory qualification. Beyond regulatory applications, the VCG concept constitutes a scalable paradigm for reducing the experimental animal use in academic and industrial research. By demonstrating how data-driven approaches can reduce animal use without compromising scientific quality, VICT3R offers an expandable model for advancing the global transition toward non-animal testing frameworks. Moreover, implementing the VCG methodology in a regulatory context could pave the way to the successful incorporation of other NAMs. The paper stresses the required paradigm shift, which involves adequate qualification procedures, cross-sector collaboration, and a cultural shift away from animal testing.

## Highlights

• Transitional strategies based on animal reduction pave the way for the phasing out of animal testing.
• Transition from concurrent to virtual control groups in animal studies significantly reduces the number of animals.
• Virtual control groups can be generated based on accumulated and curated historical data.

## 1 Introduction

Safety assessment of chemicals, cosmetics, food additives and pharmaceuticals has traditionally involved the use of animals as proxies for human physiology, exposures and responses, with sector specific regulations and guidance reflecting this paradigm. However, with the advancement of technologies using human tissue-based approaches, and the dramatic increase of data and computational tools that are now at our disposal, this paradigm is changing, with the result that testing regimes are gradually evolving to incorporate new approach methodologies (NAMs), demonstrating better translation to human outcomes and constituting potential replacements for conventional animal tests. While science has advanced rapidly, revising legislation and regulatory guidance has lagged, leading to perceptions that animal testing is necessary for regulatory submission of safety data, whereas many jurisdictions do accept that non-animal or alternative methods may provide more robust evidence of safety. The phasing out of animal testing is therefore gaining momentum worldwide, with various initiatives promoting the development and implementation of alternatives to animal testing (Beken et al., 2016; Kojima et al., 2019; EMA, 2024).

Globally, the International Council for Harmonisation of Technical Requirements for Pharmaceuticals for Human Use (ICH)^1^ supports the adoption of alternative methods for drug development where possible, while the OECD (Organisation for Economic Co-operation and Development) has worked on developing guidelines for alternative testing methods and helps to coordinate international efforts to standardize such approaches (OECD, 2005). The Food and Drug Administration (FDA) in the US is exploring alternative methods through its Implementing Alternative Methods program^2^ and announced in April 2025, a roadmap to reducing animal testing in preclinical safety studies with scientifically validated NAMs.^3^ Soon after, the National Institutes of Health (NIH), the world’s largest funder of biomedical research, announced an initiative to expand innovative, human-based science while reducing animal use in research through the establishment of an Office of Research Innovation, Validation and Application (ORIVA) .^4^ In Europe, the European Commission actively supports the development of alternatives through its Joint Research Centre^5^ and extensive funding of projects through the Framework Programmes, and in 2023 decided to phase out animal testing for chemical safety assessments in response to a European Citizens’ Initiative (European Commission, 2023a). This decision resulted in the establishment of an EU roadmap, with a plan due to be published in Q1 2026.^6^ Industries such as pharmaceutical and chemical manufacturers are also driving change, with productivity, ethical and sustainability targets requiring new ways of thinking about the development and testing of compounds, including adoption of the 3Rs principles, refinement, reduction and replacement of animal use (Harrell et al., 2024; Shenton et at., 2025).

**Fig. 1:**
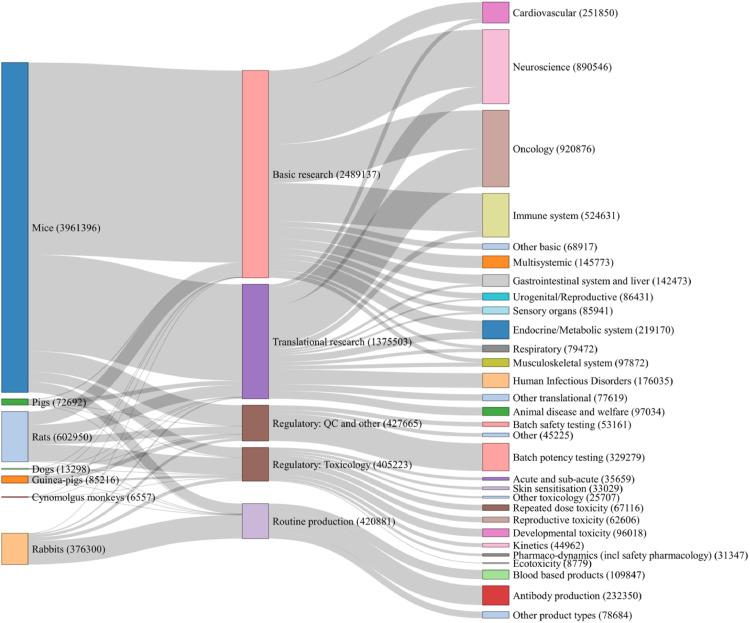
Uses of selected mammalian species in the EU, grouped by basic research, translational research, regulatory use, and routine production for year 2022. Data was retrieved from the ALURES online portal^7^ (accessed 12/03/2025).

In line with these 3Rs principles, animal use for regulatory purposes in Europe is on a significant downward trend in the recent decade (European Council, 2024). The region has the most advanced system in the world for tracking animal use, in the form of the Animal Use Reporting EU System (ALURES).^7^ This statistical database, constituted by the European Commission under directive 2010/63/EU on the protection of animals used for scientific purposes, collates information provided by the states of the European Union to generate an overview of the scientific use of animals in Europe. Comprehensive reports of ALURES are provided every 3 years. The term “use” in ALURES entails both primary use and experimental re-use of animals, while genetically altered animals are counted separately. The lack of legislation and oversight in regions other than Europe, the changes in reporting criteria, and events like underreporting or shifts from mammalian to non-mammalian uses within the region, can skew the trends of use considerably (Taylor, 2024); hence, it is advisable to look at the areas of use rather than the total uses. The uses of selected mammalian species in the EU grouped by basic research, translational research, regulatory use (quality control and toxicology) and routine production purposes for year 2022 are presented in Fig. 1. These constitute a major part of animal uses, which also to smaller extent involve aims like education or preservation of species.

According to the most recent European Commission report at the time of writing (the 2024 report on data collated from 2022), regulatory use of animals experienced important shifts particularly due to advancements in pharmaceutical, food and other legislations (European Council, 2024). Headway has been made through the departure from animal tests using replacement methods in experiments like batch release testing of vaccines and other quality testing of medicinal products, or of the mouse bioassay for shellfish toxin testing in the food sector which contributed greatly to the animal reduction in that area (European Council, 2024). However, the use of animals in basic research and translational or applied science remains the largest cause of high animal use, with the last reports showing that the research in immunology, neuroscience and oncology continue to utilize the largest amounts of animals in basic research (European Council, 2024). Though approximately 70 % of animal research is attributed to basic and translational research activities (European Commission, 2024), many current NAM approaches focus on the reduction or replacement of animals for regulatory studies due to the established standardization of study designs and related data formats, which is also the case for the VICT3R project (Steger-Hartmann et al., 2025a) discussed in the present publication. This underlines the fact that standardization of methods and data of animal experiments are key aspects for reducing the animal use (Moresis et al., 2024).

Safety testing for regulatory submissions still primarily relies on whole-animal studies focused on assessing apical endpoints of adversity. These approaches raise practical and ethical concerns, because they may lack human relevance owing to species-specific differences and may thus fail to generate mechanistic knowledge useful for predicting the development of adverse health effects in humans. To address these challenges, the beginning of the 21st century marked a paradigm shift in toxicity testing from animal-based approaches towards next generation risk assessment (NGRA) by applying NAMs. In a toxicology and chemical safety ecosystem, a NAM denotes any technology, methodology, approach or combinations of those that can provide information on chemical hazard and risk assessment in general in view of avoiding animal testing. NAMs typically, yet not uniquely, refer to *in vitro* (cell cultures and analyses), *in silico* (computational and artificial intelligence) and *in chemico* (physico-chemical properties) methods (Dent et al., 2018). In *stricto sensu*, a NAM indeed relates to the replacement component of the 3Rs principle. This explains why the NAM acronym is frequently interchangeably used to indicate new approach methodologies and non-animal methods. In a broader sense, however, a NAM could rely on lower-order *in vivo* species, in particular invertebrates, such as *Drosophilia melanogaster* (fruit fly), *Galleria mellonella* (wax moth) and *Danio rerio* (zebrafish) non-feeding embryos (Colbourne et al., 2025). Although still being debated, NAMs could equally support other pillars of the 3Rs principle, in particular initiatives revolving around reduction and refinement of animal experiments. Relevant examples in this context include short-term rodent bioassays linked with omics read-outs, which require fewer animals than the classical approaches (Schmeisser et al., 2023) and the adoption of virtual control group (VCG) concept, which may significantly reduce the number of animals used in regulatory testing (Gurjanov et al., 2024a). The potential of animal savings was first calculated by Golden et al. (2024) stating that a maximum of 25 % reduction can be achieved per study, which could even be higher in case of satellite animals used for pharmacokinetic assays or for recovery groups. In that publication, the authors estimated the minimum annual number of control animals as 15.200 (11.400 rodents and 3.800 non-rodents) used in nonclinical testing before first-in-man (*i.e.* phase 1 clinical testing) for drug applications to the FDA, which could theoretically be replaced by VCGs. However, depending on the individual study design, there will be situations where VCGs cannot be implemented, *e.g.*, usage of a new vehicle for which historical control data is not yet available. In addition, a VCG database will occasionally require new data to account for genetic drift or changes in analytical procedures. On the other hand, extending the VCG concept to other industries (*e.g.*, registration of chemicals or pesticides) will significantly increase the number of animals economized.

## 2 Initiatives to overcome NAM integration barriers

The integration of NAMs into regulatory frameworks remains slow, hindered by skepticism about *in vitro*-to-*in vivo* translation, absence of globally harmonized standards and acceptance criteria, and lack of historical precedent to guide implementation (Edwards et al., 2025; Shenton et al., 2025). The focus on the use of NAMs to create direct animal model replacements, coupled with a lack of awareness among stakeholders about the predictive accuracy and human relevance of NAMs, presents a challenge for regulatory confidence, necessitating globally harmonized validation and qualification strategies. Because NAMs are not one-to-one replacements for animal models in all contexts, integrated approaches combining several lines of evidence are essential for meeting regulatory requirements while maintaining scientific rigor and improving human relevance. Frameworks such as Integrated Approaches to Testing and Assessment (IATA) and Adverse Outcome Pathways (AOPs) facilitate the regulatory implementation of NAMs by providing a transparent rationale for their use in a safety assessment (Rovida et al., 2015; Leist et al., 2017). For example, OECD Test Guidelines for *in vitro* skin and eye corrosion/irritation rely entirely on the combination of *in chemico* and *in vitro* methods, and defined approaches for sensitization combine *in vitro* assays with computational QSAR models to predict the sensitization potential of chemicals (OECD, 2024). In parallel, AOPs for skin sensitization link molecular events, such as immune activation, to adverse outcomes, providing a mechanistic basis for interpreting NAM data and reducing reliance on animal testing (OECD, 2014).

The global regulatory landscape further complicates NAM acceptance, with significant regional variations in safety assessment requirements for the same endpoint, limiting mutual acceptance of data and hampering international harmonization, despite relevant harmonization efforts, such as the ICH and OECD mutual acceptance. Additionally, risk aversion within regulatory agencies and industry, driven by reliance on historical animal data, discourages investment in new approaches due to perceived higher costs and uncertainty. However, legacy animal data has served a constructive role by supporting the development of *in silico* predictive models (Sanz et al., 2023) and, more recently, the generation of VCGs, which offer a means to reduce animal use while maintaining a certain continuity with established procedures (Steger-Hartmann et al., 2020). VCGs make use of previously regulatory accepted animal data to create innovative computational models that simulate the expected control response in safety studies. This approach, which is the basis of the later discussed VICT3R project (Steger-Hartmann et al., 2025a), minimizes the need for dedicated control animals, reducing costs and preserving scientific rigor while aligning with ethical standards through the reduction of animal use.

Continued scientific advancement is driving the development of more predictive and human-relevant tools, accelerating the implementation of NAMs in regulatory and research contexts. In several instances, NAMs have outperformed animal models by accurately capturing human-specific biological responses. One example are liver-on-chips, which demonstrated superior predictive power for drug-induced liver injury (DILI) over traditional rodent models (Ewart et al., 2022). *In silico* models, like those within the Comprehensive *In vitro* Proarrhythmia Assay (CIPA) initiative, provide accurate predictions of proarrhythmic risk by integrating data from multiple cardiac ion channels with *in silico* electrophysiological modelling and human-derived cardiomyocyte assays, thereby enhancing translational relevance and reducing dependence on animal-based models in cardiac safety testing (Fermini et al., 2016).

In addition to scientific and regulatory hurdles, the advancement and adoption of NAMs require institutional support and consistent and well-focused funding schemes. To address this, the European Commission has implemented a broad scope of initiatives. One of these European initiatives is the Joint Research Centre’s European Union Reference Laboratory for Alternatives to Animal Testing (EURL ECVAM),^8^ established by Directive 2010/63/EU (European Union, 2010), which works on the development and promotion of animal-free approaches for testing and research purposes. Among its tasks is to validate methods (*i.e.* NAMs) that replace, reduce or refine the use of animals for the safety and efficacy/potency testing of chemicals, biologicals and vaccines. A second initiative is the European Partnership for Alternative Approaches to Animal Testing (EPAA),^9^ which is a public-private partnership between the European Commission, European trade associations and companies from seven industry sectors. EPAA commits to pool knowledge and resources to accelerate the development, validation and acceptance of animal-free approaches in regulatory testing. Another European initiative on the matter relates to funding of research and innovation on the development and use of 3Rs methods through the framework programs. In the seventh framework program (2007–2013), the European Commission spent about 200 million euro on supporting animal-free toxicology projects. A considerable part of this budget was dedicated to public-private partnerships, including the Safety Evaluation Ultimately Replacing Animal Testing consortium (SEURAT)^10^ and the Innovative Medicines Initiative (IMI),^11^ now the IHI,^12^ in which the private participation is channeled through the trade associations Cosmetics Europe and the European Federation of Pharmaceutical Industries and Associations, respectively. Moreover, the eighth framework program of the European Union (Horizon 2020; 2014–2020) includes the Animal-free Safety assessment of chemicals: Project cluster for Implementation of novel Strategies (ASPIS), which brings together three consortia funded under the European Commission’s call for projects advancing the safety assessment of chemicals without the use of animal testing. Collectively, ASPIS represents more than 70 institutions across 16 European countries and the US delivering on a €60 million investment.^13^ Regarding the NAM-related initiatives promoted by the US authorities, the NIH’s program entitled Complement Animal Research In Experimentation (Complement-ARIE)^14^ is particularly noteworthy.

## 3 Need for transitional initiatives that maintain current safety standards

Phasing out animal testing will require the replacement of existing animal assays with alternative *in silico* or *in vitro* methods, while at least safeguarding the level of consumer and patient protection achieved with the established animal assays. Despite tremendous efforts both in research capacity as well as funding allocation, the list of animal assays that can currently be replaced by *in vitro* methods is relatively short and limited to single specific endpoints, such as *in vitro* skin or eye irritation assays (OECD test guidelines 439, 437, 492) replacing the use of rabbits, the *in vitro* phototoxicity assay (OECD test guideline 432) replacing the use of albino hairless mice or guinea pigs, or the *in vitro* / *in chemico* test battery for skin sensitization (OECD test guidelines 442) replacing the use of guinea pigs. The scientific effort required to replace the guinea pig maximization test illustrates the challenges posed by more complex endpoints, which involve several key events in the AOP. Whereas the animal model agnostically represents the full AOP, including the apical outcome, namely the immunological effects of sensitization, the replacement approach requires a whole battery representing several key events (protein binding, inflammatory response, dendritic cell activation), which still lack the clinical immunological event. The latter needs to be extrapolated through computational approaches correlating the *in vitro* results with *in vivo* data.

AOPs for organ toxicities, which are usually identified in repeat dose toxicity (RDT) studies are even more complex than the mentioned AOP for skin sensitization, as was shown for liver damage (Arnesdotter et al., 2022). Detecting liver damage with AOP-based *in vitro* assays would require an even larger test battery compared to skin sensitization and would then only cover one organ. Usually, RDT studies investigate and evaluate a range of 50 to 60 organs and tissues. It is without doubt that advanced *in vitro* systems such as microphysiological systems or organ-on-chip, particularly in combination with Artificial Intelligence-based computational methods, will help to overcome this potential assay explosion based on AOPs. However, a full replacement of established systemic toxicity studies is currently not in sight. In order to bridge the gap until a total phasing out of animal studies can be realistically envisaged, transitional initiatives need to be undertaken to explore the potential to reduce animal use in conventional settings without jeopardizing the current standards of safety assessment.

The VICT3R project^15^^,^^16^ (Steger-Hartmann et al., 2025a), which is part of a series of collaborative projects aiming at reusing legacy data for improving drug safety assessment (Sanz, 2025), constitutes a clear example of a transitional initiative given that it aims to significantly reduce the number of animals used in safety assessments of drugs and other chemicals by means of the total or partial replacement of the concurrent control groups (CCGs) that are currently used with VCGs. VCGs are generated using a large database of data from control animals extracted from the archives of the companies that participate in the project (currently 20 pharmaceutical and crop science companies). The VICT3R project is supported by the industry and the European Commission through the IHI public-private partnership. The VCG concept was initially developed and prototyped during the final stages of the IMI eTRANSAFE project (Sanz et al., 2023) using data from RDT studies collected during that project. The preliminary developments and assessments of the VCG concept demonstrated its feasibility and promising performance (Gurjanov et al., 2024a; Andaya et al., 2024; Li et al., 2024). However, they also showed that additional work was required before the regulatory acceptance and routine implementation of VCGs (Palazzi et al., 2024; Sato et al., 2024), including further extension, standardization and curation of historical data from available control animals, as well as the identification of covariates that affect the VCGs (Gurjanov et al., 2023; Gurjanov et al., 2024b) and their adequate consideration during the generation of VCGs. The VICT3R project aims to address these challenges through the development, extension, validation and regulatory qualification of the VCG concept (Steger-Hartmann et al., 2025b). A key deliverable of the VICT3R project is the set-up and maintenance of an unprecedented database that contains SEND (Standard for Exchange of Nonclinical Data)^17^-standardized and curated data from animals used in regulatory safety assessments, mainly from RDT studies, and that covers a wide scope of species. The database incorporates the shared control data and allows for query and extraction of study-specific VCG data.

**Fig. 2:**
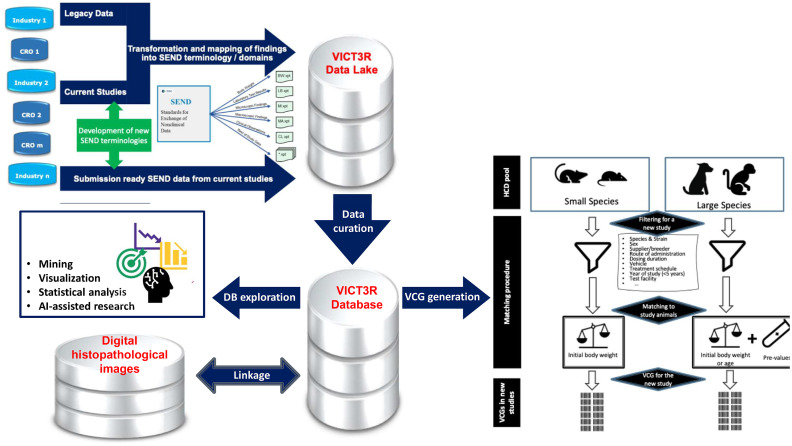
Data flows and operations in VICT3R: From raw data to VCGs.

The VICT3R consortium has developed an infrastructure for data upload, curation, sharing and querying to eventually extract appropriate VCGs from the pool of shared historical data (see Fig. 2). This infrastructure consists of a data lake where the participants of VICT3R can submit their control data in SEND format. Since the current focus of VICT3R is on RDT studies, SEND represents the key terminologies, though other standardized terminologies can be envisaged. Legacy studies, which were performed prior to SEND implementation have to be “sendified” by the donor before submitting the data. The data lake resides at an honest broker, who assures data security and preservation. Workflows have been established to curate the submitted data, (*e.g.*, checking of inconsistences, and detection of missing or duplicated data). The curated data has to be approved by the corresponding donor to assure its integrity with the raw data. Subsequently, the curated data is incorporated into the VICT3R database, which incorporate tools for data exploration, visualization and selection The database can be queried for study specific search terms, *e.g.*, species, strain, sex, route of administration, vehicle or GLP status to filter the pool of HCD relevant for the specific VCG to be generated. It is envisaged to develop tools which transfer the resulting VCGs into the laboratory-integrated management systems (LIMS) of the end user to process and analyze the VCG data together with that of the corresponding treatment groups.

Another VICT3R deliverable is the development of computational tools for the statistical characterization of the data and the generation of the VCG matching the characteristics of a particular study. Additionally, the project includes incorporation of AI-based approaches, validation of computer systems developed, assessment of the VCGs generated by means of comparative reanalysis of routine regulatory safety studies in which the CCGs are replaced by VCGs, and regulatory qualification of the new approach by regulatory agencies.

Collaborative transitional initiatives such as VICT3R help to demonstrate how secure legacy data sharing can enable the development of alternative approaches in drug and chemical safety assessment, while maintaining scientific rigor. To advance the use of alternative approaches, it is essential to gain regulatory trust and build confidence around their robustness for their recognition as safe, effective and applicable within the existing regulatory frameworks. Adopting effective step-by-step approaches helps build this trust. Rather than aiming for a complete replacement of all animal use at once, it is important to focus on targeted areas, demonstrating the viability of applying alternative approaches in these areas. This transitional strategy is being put into practice in VICT3R by proposing to replace CCGs in toxicology studies with virtual ones. Showing success in specific areas, such as this replacement of CCGs, will help to pave the way for broader adoption of non-animal approaches.

## 4 Path to regulatory acceptance of NAMs – VCGs as an example

As described above, the number of currently available animal replacement methods is still relatively low. In addition to the difficulties of replacing complex endpoints with batteries of *in vitro* tests, another reason for the low number is the considerable effort to validate the replacement methods to achieve regulatory acceptance, which is a key requirement for their use in safety and risk assessment. Validation is the process of demonstrating that a method or process consistently produces results that meet predetermined criteria. It ensures that the method is reliable and reproducible for its intended use. A validation procedure organized and performed by authorized bodies such as EURL ECVAM consists of multiple stages, including pre-validation, optimization, and inter-laboratory validation studies. The skin irritation assays validation process took 5–7 years for the individual assay types. There are no reports on the consumed financial resources for validating specifically these assays, but a recent OECD survey has come up with a minimal estimate of 0.2 to 0.5 million euro (not necessarily including manpower) for a single validation (Gourmelon et al., 2024).

A formal validation for the VCG concept is beyond the scope of the VICT3R project due to time and budget constraints. Therefore, the project envisages a qualification under the terms of the European Medicines Agency (EMA). The EMA defines qualification as a process that provides a scientific assessment of a novel methodology intended for a specific use in the context of pharmaceutical research and development. This process involves evaluating the methodology's reliability, relevance, and potential to improve drug development and regulatory decision-making (EMA 2014).

The VICT3R consortium has submitted the first request for a qualification to the EMA in 2024 for a limited context of use, namely the application of VCG in short-term animal studies used for dose-range finding (DRF). The reason for the decision to initially qualify the VCG application for only for a subset of animal studies, resulting in modest animal savings, was two-fold: these DRF studies are usually not performed under Good Laboratory Practice (GLP), *i.e.* Computer System Validation for the VCG database and the related procedures is not mandatory, reducing the validation effort. Secondly, given that the main objective of DRF studies is the identification of appropriate doses for the use in longer term animal studies and not necessarily the dose for use in humans, the risk for humans or patients which might occur through use of VCGs is significantly lower in this study type. The insights gained during the qualification for this context of use could then be used for further qualification steps covering the context of use of regulatory RDT studies performed prior to the first dosing of humans. Compared to the above-described formal validation of a NAM, the VCGs stepwise qualification for initially just one industry sector (pharmaceuticals) could represent a more cost- and time-efficient way of gradual adoption.

## 5 Opportunities for VCGs beyond regulatory assessments

**Fig. 3:**
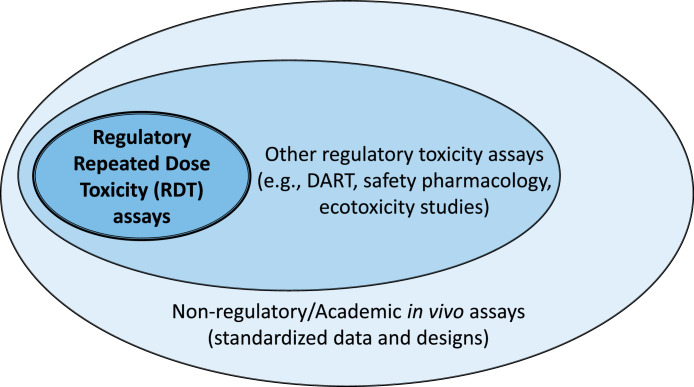
Current (RDT) and potential areas of application of the VCG concept.

Although the focus of VICT3R is on generating regulatory accepted VCGs for RDT studies, the project envisions the extension of the concept to other *in vivo* study types in both industrial and academic settings (Fig. 3). An internal survey conducted among the VICT3R industry and academic partners to explore the potential for additional regulatory studies identified developmental and reproductive toxicology (DART) studies, particularly embryofetal development studies which are the most frequent, as the most suitable candidate. The prospects for implementing VCGs in DART studies have been recently described by Wise et al. (2025). Ecotoxicity studies, which represent the second most frequent category of animal use in regulatory toxicity assessments after DART studies, as substantiated in the ALURES database, were also identified as a promising niche for VCG implementation. Quality control studies, despite representing half of the total animal use in regulatory studies, show uncertain incentives for VCG deployment, as regulatory shifts toward animal-free alternatives may soon render them obsolete (World Health Organization, 2025).

Prioritization of study types for VCG deployment considered not only the frequency each type is conducted, but for other key aspects such as consistency of the study designs, which can facilitate robust VCG generation and application. In this way, carcinogenicity studies, though less frequently performed, were identified as highly appropriate for VCG application due to their highly standardized protocols, in addition to the high amount of HCD that is available. VCG application to Safety Pharmacology (SP) *in vivo* studies is also considered generally feasible although the availability of high-quality legacy data varies across different types of SP studies. Moreover, limited information on study conditions or potential confounding variables, such as environmental factors, housing conditions and technician expertise, along with insufficient metadata, poses a significant challenge for the use of VCGs in SP studies (*e.g.* behavioral studies for CNS endpoints). Xenograft tumor models, which exhibit wide variability in experimental parameters, and *in vivo* phototoxicity assays, where protocol inconsistencies persist, have therefore been deprioritized or deemed impractical for applying the VCG concept.

The challenges for the reliable generation and use of VCGs appear to be even more pronounced in the academic context, which on the other hand is highly appealing given that most animal use occurs in basic and translational research, accounting for approximately 70 % of studies (European Commission, 2024), with the majority conducted in academic settings. The inconsistent reporting quality and the poor preservation of legacy data, an issue present in industry but further aggravated in academia (Gibney and Van Noorden, 2013; Cunha-Oliveira et al., 2024), poses an additional obstacle for assembling high-quality and consistent HCD. Moreover, the intrinsic variability of academic research is compounded by the fact that it does not typically follow standardized protocols as seen in regulatory studies. Many experimental designs are unique, with protocols frequently adapted to support innovative, hypothesis-driven research. This adaptability is not only a cornerstone of academic inquiry but also an essential component of a productive exchange, where academia often pioneers discoveries and industry integrates the resulting advances (Hait and Stoffels, 2021). Therefore, ideal conditions for VCG generation and application in academia are neither expected nor required. Still, experimental designs in certain fields exhibit a high degree of similarity, with many academic laboratories introducing only minor variations, and some phenomena being consistently observed across studies. In these cases, cross-institutional collaboration could generate a sufficiently large volume of high-quality and consistent *in vivo* data, making conceivable the generation and application of VCGs within these study types.

Despite privacy and intellectual property concerns for data sharing being present in both industry and academia, academic research generally fosters more open data sharing than industry. Open science is increasingly promoted by multiple researchers and institutions to enhance reproducibility, transparency, and collaboration. Indeed, a substantial number of datasets from academic *in vivo* research are already publicly available, supported by various initiatives, and covering new domains and data types (*e.g.* neurophysiology data, Pierré et al., 2024). This trend toward data sharing in academic research has also driven efforts to improve documentation practices. Still, a fundamental limitation hindering widespread data sharing is the lack of resources for managing and curating datasets. The VICT3R project is therefore well positioned to address these challenges and strengthen the infrastructure for data management and sharing among institutions.

The tailored use of VCGs in academic research offers significant ethical benefits while also strengthening research practices and scientific progress. The full or partial replacement of CCGs by VCGs could be particularly suited to early research phases, such as pilot and exploratory studies, whereas dual or expanded VCG approaches could improve statistical analysis in later phases (Grevot et al., 2023). Moreover, an operable collaborative database of historical datasets could offer significant benefits in terms of data exploration and analytical capabilities. It would facilitate comparative analyses to refine experimental designs and ensure methodological consistency across studies. It could also guide the selection of experimental and protocol variables, ultimately optimizing control group reliability, and therefore, making future studies more robust. Additionally, such a database could be used as a quality control or help in the identification of long-term trends and methodological artefacts that might otherwise go unnoticed. In the long run, it would enhance meta-analyses of animal studies (Hooijmans et al., 2014) and support the development of *in silico* predictive tools (Marchant et al., 2008). Overall, the standardization and sharing of academic *in vivo* data would constitute a useful approach for optimizing the planning and execution of future research, thus contributing to reducing animal numbers.

To expand the generation of databases of historical *in vivo* data to other studies beyond RDT and particularly to academic ones, the primary problem to address is the standardization that enable data reuse (Moresis et al., 2024). The first step is development of new controlled dictionaries and term mapping frameworks that align academic research reports with regulatory study reports to ensure cross-sector harmonization. In this effort, the granularity of certain variables, particularly study conditions, confounding variables and reported metadata, must be improved for both academic and regulatory research. Secondly, and building upon this, the standardization efforts must be accompanied by intensive data curation to ensure data quality. The adoption of natural language processing and collaborative curation applications, as recently done by eTRANSAFE (Corvi et al., 2025), could provide a computational support to address this issue and obtain high-value datasets enabling academic and industry researchers to upload, revise, and complement their data, addressing domain-specific challenges and reinforcing global standardization efforts for the broader implementation of VCGs.

The SEND standard is suited to providing a broad array of single subject-level data in a structured and ontologically defined format for diverse study types: single- and repeated-dose toxicity, carcinogenicity, safety pharmacology studies (respiratory and cardiovascular safety), genotoxicity (*in vivo* micro nucleus test and comet assay) and developmental and reproductive toxicology studies (currently embryo-fetal development and juvenile toxicity, with additional segments planned). Upcoming updates to the SEND standard like the nervous system domain are expected to be used to capture more findings and study types. For certain academic contexts, SEND can serve as a blueprint; in others, open data platforms, like Open Data Commons for Spinal Cord Injury (ODC-SCI) (Torres-Espín et al., 2022), provide discipline-specific, shareable data and metadata formats.

Finally, it is important to recognize that while the development of shared databases of historical data and the implementation of VCGs is technically challenging, it also requires a cultural shift in both industry and academia. Collaborative initiatives, supported by open science policies and the active involvement of scientific journals and funding agencies, are essential for fostering an environment where data sharing and standardization are prioritized. The early adoption of such practices will not only drive methodological innovation but also pave the way for broader acceptance of VCGs as a viable alternative to the CCG.

## 6 Paradigm shift toward ethical and efficient safety assessment

To advance ethical, efficient, and scientifically robust safety assessment, the systematic integration and sustained funding of NAMs must be prioritized. Achieving this requires a paradigm shift in regulatory science, one that is underpinned by targeted education, regulatory training, proactive policy engagement, and cross-sector collaboration. Efforts to enhance the responsiveness and adaptability of regulatory assessment frameworks, such as those outlined in the EMANS 2028 strategy (EMA, 2024) and by another recent publication on the matter (Edwards et al., 2025), as well as the new EMA-supported European Platform for Advancing Regulatory Science Research (Barbier et al., 2025) are critical for ensuring that evolving technologies can be effectively incorporated into decision-making processes. At the research level, consortia like NAMWISE,^18^ funded through the Horizon Europe framework, are supporting the development and validation of advanced NAMs, with a focus on human-relevant endpoints and mechanistic understanding, while also reducing animal use in line with 3Rs initiatives. Concurrently, legislative reforms such as the European Commission’s revision of the EU pharmaceutical legislation aim to establish more flexible and innovation-friendly regulatory pathways, reinforcing the role of non-animal approaches in drug development and evaluation (European Commission, 2023b).

The VICT3R project showcases successful public-private engagement, promoting structured dialogue with regulatory agencies and stakeholders, including Humane World for Animals, to advance predictive, non-animal methodologies for regulatory use. Complementing these efforts, transdisciplinary hubs, such as the Centre for Alternatives to Animal Testing in Europe (CAAT-Europe),^19^ the Centre for Documentation and Evaluation of Alternative Methods to Animal Experiments (ZEBET),^20^ and the Utrecht University Centre for Animal-Free Biomedical Translation (CPBT),^21^ play a pivotal role by providing technical guidance, training, and capacity building to facilitate the adoption of animal-free innovations across biomedical research. Taken together, these initiatives reflect a growing global commitment to transitioning toward a modernized, non-animal, human-centric safety science framework. Sustained alignment across research, policy, and regulatory domains will be critical for the systematic integration of approaches such as VCGs into regulatory frameworks.

## 7 Disclaimer

VICT3R is funded by the European Union, the private members, and those contributing partners of the IHI JU. Views and opinions expressed in this article are however those of the authors only and do not necessarily reflect those of the aforementioned parties. Neither of the aforementioned parties can be held responsible for them.

## CRediT authorship contribution statement

**Ferran Sanz:** Writing – review & editing, Writing – original draft, Supervision, Project administration, Investigation, Funding acquisition, Conceptualization. **Julia Matyjasiak:** Writing – review & editing, Writing – original draft, Visualization, Investigation, Formal analysis, Conceptualization. **Javier Orihuel:** Writing – review & editing, Writing – original draft, Investigation, Formal analysis, Conceptualization. **Colleen M. Pike:** Writing – review & editing, Writing – original draft, Investigation, Conceptualization. **Inari Soininen:** Writing – review & editing, Project administration, Funding acquisition, Conceptualization. **Frank Bringezu:** Writing – review & editing, Project administration, Funding acquisition, Conceptualization. **Olga Valverde:** Writing – review & editing, Supervision, Funding acquisition, Conceptualization. **Mathieu Vinken:** Writing – review & editing, Writing – original draft, Supervision, Investigation, Funding acquisition, Conceptualization. **Janette Turner:** Writing – review & editing, Writing – original draft, Supervision, Investigation, Conceptualization. **Thomas Steger-Hartmann:** Writing – review & editing, Writing – original draft, Supervision, Project administration, Investigation, Funding acquisition, Conceptualization.

## Declaration of competing interest

The authors declare that they have no known competing financial interests or personal relationships that could have appeared to influence the work reported in this paper.

## Data Availability

The data that was used (ALURES) is freely available at the European Commission

## Footnotes

1 https://www.ich.org/page/mission.

2 https://www.fda.gov/science-research/advancing-alternative-methods-fda/implementing-alternative-methods.

3 https://www.fda.gov/news-events/press-announcements/fda-announces-plan-phase-out-animal-testing-requirement-monoclonal-antibodies-and-other-drugs.

4 https://www.nih.gov/news-events/news-releases/nih-prioritize-human-based-research-technologies.

5 https://commission.europa.eu/about/departments-and-executive-agencies/joint-research-centre_en.

6 https://single-market-economy.ec.europa.eu/sectors/chemicals/reach/roadmap-towards-phasing-out-animal-testing_en#:∼:text=The%20European%20Commission%20is%20preparing

7 https://webgate.ec.europa.eu/envdataportal/content/alures/section2_number-of-uses.html.

8 https://joint-research-centre.ec.europa.eu/projects-and-activities/reference-and-measurement/european-union-reference-laboratories/eu-reference-laboratory-alternatives-animal-testing-eurl-ecvam_en.

9 https://single-market-economy.ec.europa.eu/sectors/chemicals/european-partnership-alternative-approaches-animal-testing_en.

10 http://www.seurat-1.eu/.

11 https://www.imi.europa.eu/.

12 https://www.ihi.europa.eu/about-ihi/imi-ihi.

13 https://aspis-cluster.eu/the-projects/.

14 https://commonfund.nih.gov/complementarie.

15 https://www.vict3r.eu/.

16 https://www.ihi.europa.eu/projects-results/project-factsheets/vict3r.

17 https://www.cdisc.org/standards/foundational/send.

18 https://ec.europa.eu/info/funding-tenders/opportunities/portal/screen/opportunities/projects-details/43108390/101191595/HORIZON.

19 https://www.biologie.uni-konstanz.de/leist/caat-europe/.

20 https://www.bfr.bund.de/en/task_of_the_zebet-58194.html.

21 https://www.uu.nl/en/news/dutch-national-growth-fund-invests-1245-million-in-transition-to-animal-free-innovation.
